# Alkylating anticancer agents and their relations to microRNAs

**DOI:** 10.20517/cdr.2019.09

**Published:** 2019-03-19

**Authors:** Bernhard Biersack

**Affiliations:** Organic Chemistry Laboratory, University of Bayreuth, Bayreuth 95440, Germany.

**Keywords:** Alkylating agents, microRNA, anticancer agents, DNA, drug resistance

## Abstract

Alkylating agents represent an important class of anticancer drugs. The occurrence and emergence of tumor resistance to the treatment with alkylating agents denotes a severe problem in the clinics. A detailed understanding of the mechanisms of activity of alkylating drugs is essential in order to overcome drug resistance. In particular, the role of non-coding microRNAs concerning alkylating drug activity and resistance in various cancers is highlighted in this review. Both synthetic and natural alkylating agents, which are approved for cancer therapy, are discussed concerning their interplay with microRNAs.

## Introduction

Alkylating agents represent an important tool for the daily fight against cancer. Interestingly, their introduction into clinical application based on chemical warfare. Like a knife that can kill in the hands of an assassin and heal in the hands of a surgeon, the potential of nitrogen and sulfur mustards as anticancer agents was identified after incidents with poison gas (reduction of white blood cells) during World War II and dates back to observations after poison gas attacks during World War I^[1,2]^. Rational chemical modifications led to less lethal anticancer drug candidates such as melphalan, chlorambucil, and cyclophosphamide (all of them are nitrogen mustards). Alkylation of bionucleophiles (proteins, nucleic acids) mainly occurs at *N*, *O*, and *S* sites with free electron pairs. In most cases DNA was identified as the main target of alkylating agents and both mono-functional (reaction with only one DNA strand) and bifunctional alkylating agents (reaction with two strands leading to DNA crosslinks) are known^[2]^. Pertinent drug resistance strategies of cancers in order to cope with alkylating drugs include elevated glutathione levels, enhanced DNA repair and modified DNA damage signaling, as well as expression of multidrug resistance proteins such as membrane transporters^[2]^. The prodrug temozolomide is a highlight of research concerning alkylating agents because it can penetrate the blood-brain-barrier and is clinically applied for the therapy of glioblastoma^[3]^. Meanwhile, nature has also provided very potent alkylating agents with promising anticancer activities such as mitomycins, cyclopropyl-indoles, and illudins^[4-6]^. Mitomycin C is applied for the treatment of various solid tumors^[4]^. Trabectedin is the latest natural alkylating agent that has been approved for the therapy of soft tissue sarcoma and platinum-resistant ovarian cancer^[7]^.

MicroRNAs (miRNAs) are small RNA molecules of 22-23 nucleotides and regulate numerous genes involved in cell differentiation, cell proliferation, and cell death^[8]^. Mature miRNAs usually bind to the 3’-untranslated region of target messenger RNAs (mRNAs) and one miRNA is able to bind to various mRNAs^[9-11]^. MiRNAs have become valuable tools for the diagnosis and prognosis of cancer diseases due to abnormal expression profiles in cancers^[12,13]^. In addition, miRNAs regulate metastasis formation (epithelial-to-mesenchymal transition) and survival of cancer stem-like cells^[14,15]^. Concerning cancer research, tumor suppressor miRNAs and oncogenic miRNAs (oncomirs) are of particular interest and played crucial roles for the sensitivity and resistance of various tumors to applied drugs^[16]^. Prominent miRNAs with great relevance to cancer disease are represented by the tumor suppressor let-7 family and the oncomir miR-21^[17,18]^.

This review intends to give an overview of clinically approved alkylating agents and their interactions with miRNAs in cancer diseases concerning drug activity and resistance.

## Synthetic alkylating agents and their interactions with miRNAs

Synthetic alkylating agents are widely applied for the therapy of solid tumors and of leukemia/lymphoma diseases. Relevant synthetic alkylating agents that are dealt with in this review can be subdivided into the following compound classes: mustards (e.g., nitrogen mustards such as mechlorethamine, melphalan, chlorambucil, bendamustine, cyclophosphamide, estramustine), diazomethane forming prodrugs (e.g., dacarbazine, temozolomide), and *N*-nitrosoureas (e.g., BCNU/carmustine). The influence of miRNAs on the activity of these alkylating drugs is discussed below.

## Nitrogen mustards, miRNAs and cancer

**Figure 1:**
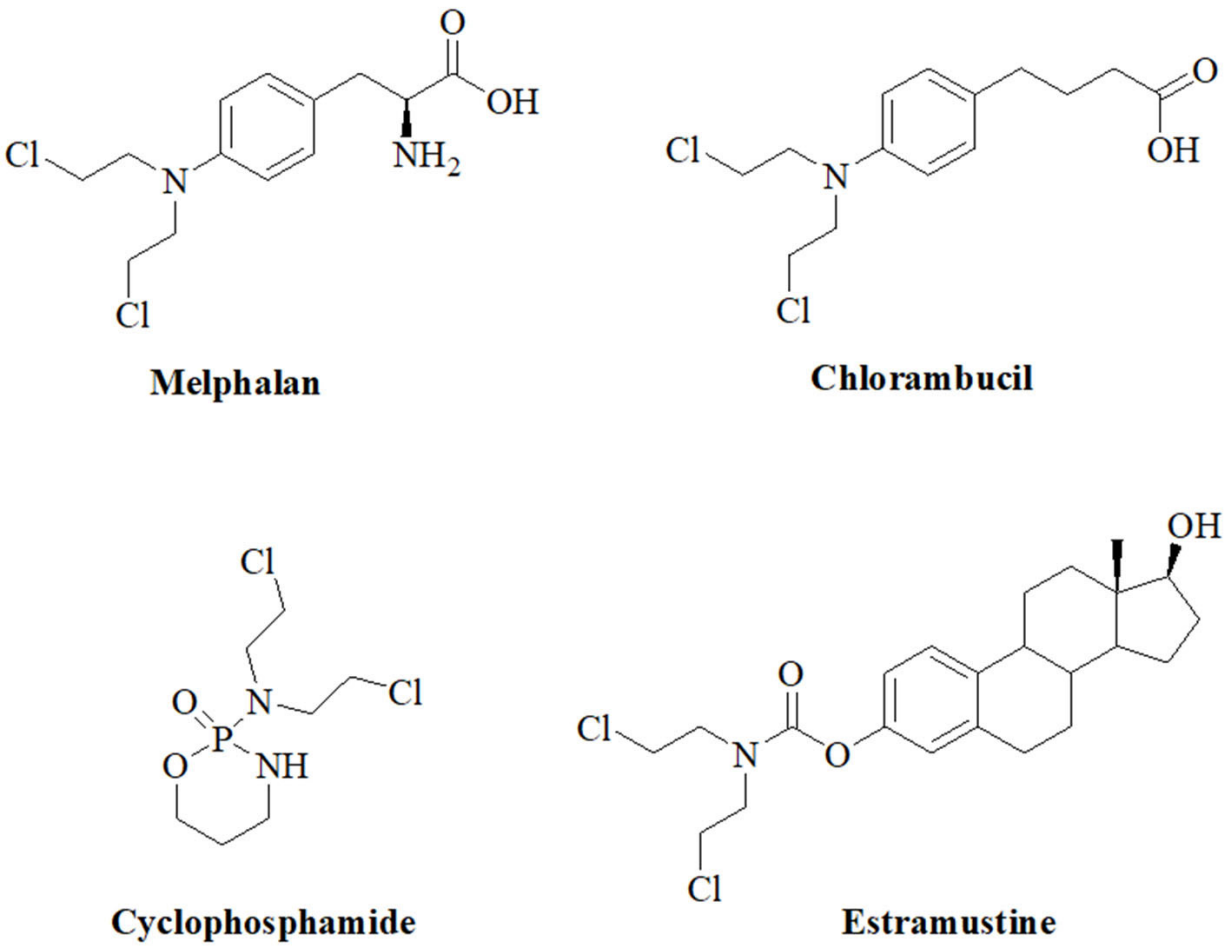
Structures of the *N*-mustard alkylating agents melphalan, chlorambucil, cyclophosphamide and estramustine

As mentioned above, anticancer active nitrogen mustards were developed since 1942 from highly toxic poison gas applied or produced in both World Wars of the 20th century. In order to reduce the systemic toxicity of nitrogen mustard poison gas and of initially applied mustard drugs like mechlorethamine, anilin derivatives such as chlorambucil and melphalan were designed with reduced activity due to their aromatic amine system [Figure 1]. Soon later, bendamustine and the prodrug cyclophosphamide were developed as potent anticancer drugs [Figure 1]^[19]^. In addition, the alkylating estrogen-conjugate estramustine was disclosed [Figure 1]^[19]^. Recent efforts in the field of nitrogen mustard-based anticancer research to increase selectivity and reduce side effects included DNA-targeting strategies, brain-targeting strategies, antibody-directed enzyme prodrug therapy and gene-directed enzyme prodrug therapy strategies, and nitrogen mustard prodrugs activated by glutathione transferase^[19]^. The alkylation mode of action of these 2-chloroethylamino derivatives includes an intramolecular reaction to an aziridium intermediate that readily reacts with bionucleophiles^[19]^. Aside cytotoxic effects on non-malignant cells, genotoxic mutagenic effects were identified for nitrogen mustards as well.

Chlorambucil has been the standard treatment for chronic lymphocytic leukemia (CLL) for decades and the drug is still recommended as a mainstay for the currently widely applied antibody-based chemoimmunotherapy for CLL^[20]^. Chemoresistance of CLL is often mediated by the p53-signaling pathway which plays a crucial role for the cellular response to DNA damage^[21]^. The tumor suppressor microRNA miR-34a is connected with p53-signaling and low expression of miR-34a led to chemoresistance in CLL^[22]^. A considerable number of PCLBCL-LT (primary cutaneous diffuse large B-cell lymphomas, leg-type) patients did not respond to chlorambucil first-line therapy and upregulated expression of the oncogenic miR-17-92 cluster seems responsible leading to suppression of PTEN (phosphatase and TENsin homolog)^[23]^. A miR-210 targeting/inhibiting chlorambucil conjugate was synthesized, which suppressed a cancer relevant hypoxic mechanism and was active against hypoxic triple-negative breast cancer in mice^[24]^. A list of miRNAs associated with chlorambucil activity is provided in Table 1.

**Table 1:** MiRNAs with effects on the anticancer activity of chlorambucil

MiRNA	Target	Function	Expression in cancers/tissues
miR-34a	p53	Tumor suppressor	Suppressed in resistant CLL
miR-17-92 cluster	PTEN	Oncomir	Upregulated in resistant B-cell lymphoma
miR-210	GPD1L	Oncomir	Upregulated in triple-negative breast cancer

CLL: chronic lymphocytic leukemia; GPD1L: glycerol-3-phosphate dehydrogenase 1L; PTEN: phosphatase and TENsin homolog

Melphalan is a close analog of chlorambucil and has been applied for the treatment of multiple myeloma (MM) since the 1960’s^[25]^. From MM patients treated with melphalan circulating exosomal let-7b and miR-18a were identified as prognostic factors (i.e., improved survival)^[26]^. Suppression of the oncogenes Myc, Ras and CCND1 (cyclin D1) by let-7b may play a role as well as inhibition of the HIF1α-pathway [hypoxia-inducible factor (HIF)] by miR-18a^[26]^. IL-6-mediated downregulation of the tumor suppressor miR-15a/16 expression increased resistance of myeloma cells to melphalan treatment^[27]^. The oncogenic miR-221-222 family suppressed p53 upregulated modulator of apoptosis (PUMA) in MM cells leading to drug resistance^[28]^. Inhibition of miR-221 by a 13 mer LNA-i-miR-221 inhibitor broke melphalan resistance in MM by modulation of PUMA (upregulation) and ATP binding cassette C1 (ABCC1) transporter (downregulation)^[29]^. In addition, it was suggested that suppression of miR-451 can enhance the activity of melphalan in multiple myeloma via downregulation of multidrug resistance gene 1 (MDR1)^[30]^. A list of miRNAs involved in melphalan activity in myeloma is given in Table 2.

**Table 2:** MiRNAs with effects on the anticancer activity of melphalan

MiRNA	Target(s)	Function	Expression in cancers/tissues
let-7b	Myc, Ras, CCND1	Tumor suppressor	Exosomal let-7b upregulated in sensitive MM
miR-15a/16	-	Tumor suppressor	Suppressed in resistant myeloma
miR-18a	HIF1α	Tumor suppressor	Exosomal miR-18a upregulated in sensitive MM
miR-221-222 family	PUMA, ABCC1	Oncomir	Upregulated in resistant MM
miR-451	MDR1	Oncomir	Suppressed in resistant MM

ABCC1: ATP-binding cassette C1; CCND1: cyclin D1; HIF1α: hypoxia-inducible facter 1-α; MDR1: multidrug resistance gene 1; MM: multiple myeloma; PUMA: p53 upregulated modulator of apoptosis

Cyclophosphamide is one of the most successful anticancer drugs which is still widely applied for the therapy of many cancer diseases 60 years now after its development in the late 1950’s^[31]^. The drug is applied for the treatment of breast cancer, childhood cancers, and lymphoma^[31]^. Cyclophosphamide is a prodrug, which can be activated in a chemical or metabolic way. Enzymatic oxidation of cyclophosphamide generates cytotoxic phosphoramide mustard molecules leading to interstrand and intrastrand crosslinks of DNA as well as toxic acrolein which is responsible for the side effects of cyclophosphamide^[32]^.

Clinical sensitivity to cyclophosphamide is strongly correlated with the ability of cancer cells to induce apoptosis upon DNA damage^[33]^. Due to its significance in cancer therapy the relations between miRNAs and cyclophosphamide is well studied, in particular, in lymphoma^[34,35]^. Upregulation of circulating oncogenic miR-125b and miR-130a was determined in B-cell lymphoma samples from patients treated with cyclophosphamide-based chemotherapy^[36]^. In diffuse large B-cell lymphoma (DLBCL, the most common non-Hodgkin lymphoma type) treated with R-CHOP (rituximab, cyclophosphamide, adriamycin, vincristine, and prednisone), increased miR-181a expression prolonged progression free survival (PFS) by suppression of FOXP1 and *O*6-methylguanine DNA methyltransferase (MGMT) while increased miR-18a levels led to shorter overall survival (OS) and high miR-222 expression to shorter PFS^[37]^. MiR-93 (targets: p21, BCL2L11), miR-221 and miR-222 (target: p27) were upregulated in DLBCL patients with poor outcomes after cyclophosphamide-based therapies^[38]^. Knockdown of the well-known oncomir miR-21 sensitized DLBCL cells to CHOP treatment by upregulation of PTEN^[39]^. Shorter OS was observed from DLBCL patients with high miR-21 expression in the tumor tissue, while high miR-21 levels in the serum promoted relapse free survival^[40]^. Downregulated miR-199a/b also shortened progression free survival time^[40]^. In addition, CHOP or R-CHOP treated DLBCL patients with upregulated miR-200c expression displayed shorter OS than patients with suppressed miR-200c^[41]^. Induced miR-155 expression was connected with R-CHOP resistance in DLBCL patients although miR-155 sensitized patients to AKT (protein kinase B, ak thymoma) signaling probably by targeting p85α and SHIP1^[42]^. In contrast to that, upregulated expression of miR-129-5p in DLBCL patients treated with CHOP or R-CHOP led to much longer median survival than in patients with downregulated miR-129-5p^[43]^. The tumor suppressor miR-146a was downregulated in R-CHOP-treated DBCL patients who displayed drug resistance^[44]^. Suppressed expression of the tumor suppressors miR-146b-5p and miR-320d led to shorter progression free survival in CHOP-treated DLBCL patients^[45]^. Higher serum levels of miR-33a and miR-455-3p indicated better response while high levels of miR-224 (target: CD59), miR-520d-3p and miR-1236 were connected with worse R-CHOP response in DLBCL patients^[35,46]^. Suppressed miR-224 expression in the tumor tissue also indicated poor survival^[39]^. In addition, a higher R-CHOP response rate and longer survival was observed from DLBCL patients with 7q gain, which was attributed to the upregulated expression of miR-25, miR-96, miR-182, and miR-589^[47]^. Lists of miRNAs involved in cyclophosphamide activity in lymphoma are given in Tables 3 and 4.

**Table 3:** Effects of oncomirs on the anti-lymphoma activity of cyclophosphamide

MiRNA	Expression (targets)
miR-18	Upregulated in patients with shorter OS
miR-21	Upregulated in tumor cells and tissues leading to shorter OS (PTEN)
miR-93	Upregulated in resistant patients (p21, BCL2L11)
miR-125b	Upregulated in patients treated with R-CHOP
miR-130a	Upregulated in patients treated with R-CHOP
miR-155	Upregulated in R-CHOP-resistant patients (p85α, SHIP1)
miR-200c	Upregulated in patients with shorter survival
miR-221	Upregulated in resistant patients (p27)
miR-222	Upregulated in resistant patients with shorter PFS (p27)
miR-224	Expression in serum connected with worse R-CHOP response
miR-520d-3p	Expression connected with worse R-CHOP response
miR-1236	Expression connected with worse R-CHOP response

OS: overall survival; BCL2L11: Bcl2-like protein 11; PTEN: phosphatase and tensin homolog; R-CHOP: rituximab plus cyclophosphamide, hydroxydaunorubicin (doxorubicin), oncovin (vincristine), prednisone; SHIP1: SH2 domain-containing inositol phosphatase 1; PFS: progression free survival

**Table 4:** Effects of tumor suppressing miRNAs on the anti-lymphoma activity of cyclophosphamide

MiRNA	Expression (targets)
miR-21	Upregulation in serum promotes survival
miR-25	Expression connected with longer survival in 7q gain patients
miR-33a	Expression connected with better R-CHOP response
miR-96	Expression connected with longer survival in 7q gain patients
miR-129-5p	Upregulated in patients with longer median survival
miR-146a	Suppressed in R-CHOP-resistant patients
miR-146b-5p	Suppressed in patients with shorter PFS
miR-181a	Upregulated in patients with prolonged PFS (FOXP1, MGMT)
miR-182	Expression connected with longer survival in 7q gain patients
miR-199a/b	Suppression connected with shorter PFS
miR-224	Suppression in tumor tissue connected with poor survival
miR-320d	Suppressed in patients with shorter PFS
miR-455-3p	Expression connected with better R-CHOP response
miR-589	Expression connected with longer survival in 7q gain patients

FOXP1: forkhead box protein P1; MGMT: O6-methylguanine DNA methyltransferase; R-CHOP: rituximab plus cyclophosphamide, hydroxydaunorubicin (doxorubicin), oncovin (vincristine), prednisone; PFS: progression free survival

MicroRNAs also seem to play a role for drug resistance in breast cancer patients receiving cyclophosphamide as part of the TFAC therapy (paclitaxel, 5-fluorouracil, adriamycin, cyclophosphamide)^[48]^. The tumor suppressor miR-9 bound to the mRNA of human epidermal growth factor 2 (HER2) and increased the response to cyclophosphamide^[49]^. Low expression of let-7, miR-10b, miR-34, miR-155, miR-200c, miR-205, miR-451, and miR-3200 as well as high expression of miR-21, miR-195, and miR-221 were observed after cyclophosphamide treatment^[50,51]^. In particular, expression of the tumor suppressor miR-205 sensitized breast cancer cells to TAC treatment (docetaxel, doxorubicin, cyclophosphamide) by suppression of vascular endothelial growth factor A and fibroblast growth factor 2^[52]^. In contrast to that, miR-663 overexpression was associated with resistance to cyclophosphamide in MDA-MB-231/ADM breast cancer cells (cells resistant to adriamycin) by suppression of heparin sulfate proteoglycan 2^[53]^. The MDA-MB-231 cell line is a widely applied model for triple-negative breast cancer (TNBC, i.e., no or reduced expression of estrogen receptor, HER2/neu and progesterone receptor), which is a very problematic form of breast cancer showing chemotherapy resistance and poor prognosis. Samples from cyclophosphamide-treated TNBC patients with good response to chemotherapy exhibited higher miR-200b-3p (possible targets: PLCB1, MYCN, CCND2, RERG) and miR-190a (possible targets: BCL11A, CALCR, FOXP2, HOXC5) as well as lower miR-512-5p (possible targets: BCL2L2, POLD3, c-Myc) expression when compared with TNBC patients displaying weak chemotherapy response^[54]^. The microRNAs miR-30a, miR-9-3p, miR-770 and miR-143-5p were also identified as markers for chemotherapy response by TNBC patients. Chemotherapy-responding TNBC patients revealed upregulated miR-30a (affected transcriptional regulation in cancer) and miR-9-3p (affected mTOR/mammalian target of rapamycin and TGF/transforming growth factor-β signaling) as well as suppressed miR-770-5p (affected B and T cell receptor signaling) and miR-143-5p (affected B and T cell receptor signaling as well as mTOR signaling)^[55]^. A list of miRNAs involved in cyclophosphamide activity in breast cancer is given in Table 5.

**Table 5:** MiRNAs with effects on the anti-breast cancer activity of cyclophosphamide

MiRNA	Target(s)	Function	Expression
let-7	-	Tumor suppressor	Low expression upon CP treatment
miR-9/miR-9-3p	HER2, mTOR and TGF-β signaling	Tumor suppressor	Expression increased response to CP
miR-10b	-	Tumor suppressor	Low expression upon CP treatment
miR-21	PTEN	Oncomir	High expression upon CP treatment
miR-30a	transcriptional regulation	Tumor suppressor	Upregulation connected with better response
miR-34	-	Tumor suppressor	Low expression upon CP treatment
miR-143-5p	B and T cell receptor and mTOR signaling	Oncomir	Suppression connected with better response
miR-190a	BCL11A, CALCR, FOXP2, HOXC5	Tumor suppressor	High expression correlated with better response
miR-195	-	Oncomir	High expression upon CP treatment
miR-200b-3p	PLCB1, MYCN, CCND2, RERG	Tumor suppressor	High expression correlated with better response
miR-200c	-	Tumor suppressor	Low expression upon CP treatment
miR-205	VEGFA, FGF2	Tumor suppressor	Low expression upon CP treatment, expression sensitizes to TAC treatment
miR-221	PUMA, ABCC1	Oncomir	High expression upon CP treatment
miR-451	-	Tumor suppressor	Low expression upon CP treatment
miR-512-5p	BCL2L2, POLD3, c-Myc	Oncomir	Suppression correlated with better response
miR-663	HSPG2	Oncomir	Expression connected with resistance of cancer cells
miR-770-5p	B and T cell receptor signaling	Oncomir	Suppression connected with better response
miR-3200	-	Tumor suppressor	Low expression upon CP treatment

ABCC1: ATP-bonding cassette C1; BCL11A: B-cell lymphoma/leukemia 11A; BCL2L2: Bcl-2-like protein 2; CALCR: calcitonin receptor; CCND2: cyclin D2; CP: cyclophosphamide; FGF2: fibroblast growth factor 2; FOXP2: forkhead-box-protein P2; HER2: human epidermal growth factor receptor 2; HSPG2: heparin sulfate proteoglycan 2; mTOR: mammalian target of rapamycin; MYCN: gene coding for N-Myc; POLD3: DNA polymerase delta 3; PTEN: phosphatase and TENsin homolog; PUMA: p53 upregulated modulator of apoptosis; RERG: RAS-like estrogen-regulated growth inhibitor; TAC: docetaxel, doxorubicin, cyclophosphamide; TGF-β: transforming growth factor β; VEGFA: vascular endothelial growth factor A

The relations between miRNAs and cyclophosphamide were also investigated in further cancer diseases. VAC treatment (vindesine, doxorubicin, cyclophosphamide) is applied as a second-line treatment of small cell lung cancer. The structurally simple HDAC inhibitor valproic acid increased the activity of VAC treatment both *in vitro* and *in vivo* via induction of miR-589 and suppression of miR-575 expression^[56]^. In addition, overexpression of the oncomirs miR-27b and miR-298 led to cyclophosphamide resistance in Panc-1 pancreatic cancer cells by inhibition of vitamin D receptor and cytochrome P3A4 (CYP3A4)^[57]^. It became evident that CYP3A4 plays a crucial role for the activation of cyclophosphamide in these cancer cells. A list of miRNAs involved in cyclophosphamide activity in various cancers is given in Table 6.

**Table 6:** MiRNAs with effects on the activity of cyclophosphamide in miscellaneous cancers

MiRNA	Target	Function	Expression in cancers/tissues
miR-27b	CYP3A4, VDR	Oncomir	Overexpression in resistant pancreas cancer cells
miR-298	CYP3A4, VDR	Oncomir	Overexpression in resistant pancreas cancer cells
miR-575	Invasion	Oncomir	Suppression led to sensitive lung cancer cells
miR-589	EMT	Tumor suppressor	High expression led to sensitive lung cancer cells

CYP3A4: cytochrome P3A4; EMT: epithelial-to-mesenchymal transition; VDR: vitamin D receptor

Estramustine phosphate is applied for the treatment of advanced prostate cancer and combines the DNA-damaging *N*-mustard scaffold with a tubulin polymerization inhibiting steroid (estradiol-17β-phosphate) released upon metabolization of the drug^[58]^. In prostate cancer cells, estramustine phosphate induced apoptosis via suppression of the oncomir miR-31^[59]^. In addition, downregulation of the tumor suppressor miR-4319, which suppresses HER2 expression, was associated with poor chemotherapy response in prostate cancer patients and induced miR-4319 expression sensitized prostate cancer cells to estramustine treatment^[60]^. A list of microRNAs involved in estramustine phosphate anticancer activity is given in Table 7.

**Table 7:** MiRNAs with effects on the anticancer activity of estramustine phosphate

MiRNA	Target	Function	Expression in cancers/tissues
miR-31	Apoptosis	Oncomir	Suppression in prostate cancer led to apoptosis
miR-4319	HER2	Tumor suppressor	Suppression in prostate cancer patients led to resistance

HER2: human epidermal growth factor receptor 2

## Dacarbazine and temozolomide, miRNAs and cancer

**Figure 2:**
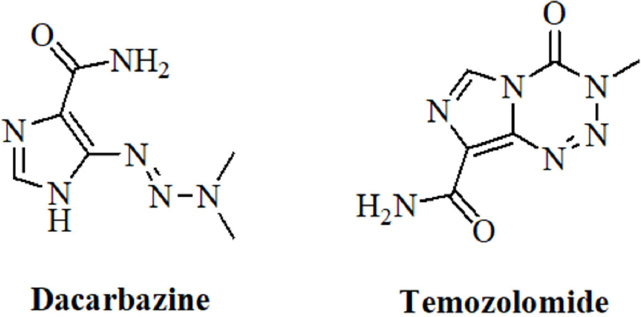
Structures of the alkylating agents dacarbazine and temozolomide

Dacarbazine and temozolomide [Figure 2] are valuable anticancer drugs for the treatment of melanoma and of brain tumors such as glioblastomas (GBMs)^[61,62]^. Their mode of action implies the generation of highly toxic DNA-alkylating diazomethane molecules that kill the cancer cells^[63]^.

Growth inhibition of melanoma cells by dacarbazine was mediated by induction of the expression of miR-200 family miRNAs (miR-141, miR-200a/b/c) which regulate apoptosis^[64]^. Dacarbazine is also widely applied for the treatment of lymphomas as part of the ABVD therapy (adriamycin, bleomycin, vinblastine, dacarbazine). Complete response to ABVD therapy was achieved by classical Hodgkin lymphoma patients with reduced miR-494 and miR-1973 plasma levels^[65]^. A list of miRNAs involved in dacarbazine activity in various cancers is given in Table 8.

**Table 8:** MiRNAs with effects on the anticancer activity of dacarbazine

MiRNA	Target	Function	Expression in cancers/tissues
miR-141	Apoptosis	Tumor suppressor	Expression sensitized melanoma cells
miR-200a/b/c	Apoptosis	Tumor suppressor	Expression sensitized melanoma cells
miR-494	-	Oncomir	Suppression led to complete response in lymphoma patients
miR-1973	-	Oncomir	Suppression led to complete response in lymphoma patients

The development of temozolomide as an anticancer drug started in the 1980’s and is a nice example of the productive interplay between chemists, pharmacists and clinicians during the development process^[63]^. Temozolomide is a prodrug and decomposes in aqueous solution. Initially CO_2_ is released from the molecule whereupon diazomethane is formed that reacts with bionucleophiles^[63]^. The interplay between temozolomide and miRNAs in cancer was thoroughly studied over the last years. Initial studies revealed that temozolomide-induced apoptosis was inhibited by upregulated miR-21 accompanied by decreased Bax/Bcl-2 ratio and caspase-3 activity in GBM cells^[66,67]^. Treatment of GBM cells with temozolomide led to increased miR-21 expression and GBM cells were sensitized to temozolomide by downregulation of miR-21 and miR-17^[68-70]^. In particular, antisense miR-21 formulated with PLGA [poly(lactic-co-glycolic acid)] nanoparticle carriers increased the anticancer activity of temozolomide against GBM cells^[71]^. Temozolomide-resistant GBM cells also exhibited increased miR-10a*, miR-195 (also in cancer stem cells of GMB tumors via mothers against decapentaplegic homolog 2 targeting), and miR-455-3p expression^[72,73]^. However, in melanoma cells, miR-195 functioned as a tumor suppressor by suppression of prohibitin 1^[74]^. In addition, chemoresistance to temozolomide was associated with upregulation of miR-9 (targeting/patched homolog 1) in CD133-positive GBM cells and of Bcl-2-interacting mediator of cell death - targeting miR-138^[75-77]^. Downregulation of the tumor suppressor miR-16 increased Bcl-2 activity and led to temozolomide resistance in glioma cells^[78]^. Suppressed miR-29c expression associated with upregulated reversionless 3-like (REV3L) led to temozolomide resistance in glioma cells^[79]^. In addition, miR-29c enhanced temozolomide activity by indirect suppression of MGMT^[80]^. MiR-30a expression also enhanced temozolomide activity against U251 GBM cells by inhibition of autophagy and suppression of beclin 1^[81]^. Upregulation of the tumor suppressor miR-136 led to higher temozolomide activity in glioma cells by suppression of astrocyte elevated gene 1 (AEG-1)^[82]^. Similarly, induced miR-143 expression increased temozolomide activity against GBM cells via neuroblastoma rat sarcoma oncogene targeting^[83]^. The oncomir miR-93 led to temozolomide resistance of glioma cells by upregulation of p21^[84]^. MiR-125b-2 induced temozolomide resistance in glioblastoma stem cells was associated with downregulation of Bax and upregulation of Bcl-2^[85]^. Downregulation of the oncomirs miR-221/miR-222 refurnished p53 signaling pathway and promoted apoptosis of GBM cells treated with temozolomide^[86]^. MiR-141-3p is another p53-targeting oncomir leading to temozolomide resistance in glioma cells^[87]^. MAP kinase/extracellular-signal regulated kinase (ERK) signaling was induced by the oncomir miR-299-5p, which targeted golgi phosphoprotein 3 and, thus, led to temozolomide resistance in GBM cells^[88]^. Temozolomide resistance in GBM basing on active GSK3β was overcome by upregulated miR-101^[89]^. Further to this, miR-128 mediated temozolomide-induced cell death in glioma cells via inhibition of mTOR signaling and suppression of insulin-like growth factor 1, phosphoinositide-3-kinase regulatory subunit 1, rapamycin-insensitive companion of mTOR and mTOR^[90]^. High expression of miR-130a sensitized glioma cells to temozolomide via apurinic/apyrimidinic endonuclease 1 suppression and was upregulated in GBM patients with better response to temozolomide^[91]^. A direct influence on temozolomide resistance was elucidated for miR-181d and miR-603, which suppressed the DNA repair enzyme MGMT leading to greater sensitivity to temozolomide^[92]^. In addition, miR-142-3p promoted temozolomide activity in GBM cells by suppression of MGMT^[93]^. MiR-648 and miR-767-3p were identified as further MGMT targeting/suppressing miRNAs, which enhanced the anticancer activity of temozolomide in T98G GBM cells^[94]^. MiR-182 expression also increased temozolomide activity in GBM cells by apoptosis promotion via c-Met, HIF2A and BCL2L12 suppression^[95]^. However, expression of miR-132 caused temozolomide resistance in GBM cells (U78MG) via downregulation of tumor suppressor candidate 3^[96]^. Temozolomide-resistant GBM cells and tissues exhibited suppressed miR-370-3p expression levels, which is a tumor suppressor responsible for downregulated MGMT expression and blocked DNA repair^[97]^. In contrast to that, expression of the oncomir miR-423-5p led to temozolomide resistance in glioma cells by targeting ING-4^[98]^. Glioblastoma samples from patients treated with temozolomide revealed upregulated miR-629-3p expression (targeting genes involved in translation and RNA processing) in case of good responders with prolonged OS^[99]^. The tumor suppressor miR-1268 also sensitized glioma cells (T98G) to temozolomide treatment^[100]^. In addition, miR-1294 suppressed targeting protein for Xenopus kinesin-like protein 2 in glioma cells leading to enhanced temozolomide activity^[101]^. Some miRNAs are special “Janus-type” cases here. Although miR-181b/c are reported as tumor suppressors in GBM, reduced expression of these miRNAs was associated with better temozolomide response^[102]^. And although miR-221 and miR-222 suppress MGMT, their upregulation led to weaker responses to temozolomide^[103]^. In a cancer type dependent way, miR-195 acted either as a tumor suppressor (melanoma) or as an oncomir (GBM, see above). Last but not least, the combination of temozolomide with miRNA-regulating drugs appears promising. For instance, curcumin (a polyphenol isolated from turmeric, *Curcuma longa*) was able to sensitize GBM cells (C6) to temozolomide via suppression of miR-10b^[104]^. Lists of miRNAs involved in temozolomide anticancer activity are given in Tables 9 and 10.

**Table 9:** Tumor suppressing miRNAs with effects on the anticancer activity of temozolomide

MiRNA	Target(s)	Expression in cancers/tissues
miR-16	Bcl-2	Suppression in GBM led to resistance
miR-29c	MGMT, REV3L	Suppression in glioma led to resistance
miR-30a	Beclin 1	High expression sensitized glioma cells
miR-101	GSK3β	High expression sensitized GBMs
miR-128	IGF1, PIK3R1, RICTOR, mTOR	Expression sensitized glioma cells
miR-130a	APE1	High expression sensitized glioma cells
miR-136	AEG-1	High expression sensitized glioma cells
miR-142-3p	MGMT	Expression sensitized GBM
miR-143	N-RAS	High expression sensitized GBM
miR-181d	MGMT	Expression sensitized GBM
miR-182	c-MET, HIF2A, BCL2L12	High expression sensitized GBM
miR-195	PHB1	Expression sensitized melanoma
miR-370-3p	MGMT	Suppression in resistant GBM
miR-603	MGMT	High expression sensitized GBM
miR-629-3p	Translation	High expression in GBM prolonged OS
miR-1268		Expression sensitized glioma cells
miR-1294	TPX2	Expression sensitized glioma cells

AEG-1: astrocyte elevated gene-1; APE1: apurinic/apyrimidinic endonuclease 1; Bcl-2: B-cell lymphoma 2; GSK3β: glycogen synthase kinase 3β; IGF1: insulin-like growth factor 1; MGMT: O6-methylguanine-DNA methyltransferase; mTOR: mammalian target of rapamycin; N-RAS: neuroblastoma rat sarcoma oncogene; PHB1: prohibitin 1; PIK3R1: phosphoinositide-3-kinase regulatory subunit 1; REV3L: reversionless 3-like; RICTOR: rapamycin-insensitive companion of mTOR; TPX2: targeting protein for Xenopus kinesin-like protein 2

**Table 10:** Oncomirs with effects on the anticancer activity of temozolomide

MiRNA	Target(s)	Expression in cancers/tissues
miR-9	PTCH1	High expression in resistant GBM
miR-10*	-	High expression in resistant GBM
miR-10b	-	Suppression sensitized GBM cells
miR-17	ATG7	Suppression sensitized GBM cells
miR-21	Bax/Bcl2	Suppression sensitized GBM cells
miR-93	p21	Expression correlated with resistance in glioma cells
miR-125b-2	Bax	Expression correlated with glioblastoma stem cell resistance
miR-132	TUSC3	Expression correlated with resistance in GBM cells
miR-138	BIM	High expression in resistant GBM
miR-141-3p	p53	Expression correlated with resistance in glioma cells
miR-195	-	High expression in resistant GBM
miR-221	p53	Suppression promotes apoptosis in GBM cells
miR-222	p53	Suppression promotes apoptosis in GBM cells
miR-299-5p	GOLPH3	Expression correlated with resistance in GBM cells
miR-423-5p	ING-4	Expression correlated with resistance in glioma cells
miR-455-3p	SMAD2	High expression in resistant GBM

ATG7: autophagy-related protein 7; Bax: Bcl-2-associated X protein; Bcl-2: B-cell lymphoma 2; BIM: BCL-2-interacting mediator of cell death; GOLPH3: golgi phosphoprotein 3; ING-4: inhibitor of growth protein 4; PTCH1: patched homolog 1; SMAD2: mothers against decapentaplegic homolog 2; TUSC3: tumor suppressor candidate 3

### *N*-nitrosoureas, miRNAs and cancer

**Figure 3:**
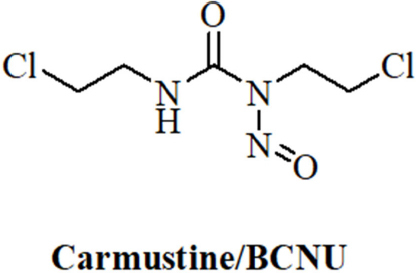
Structure of the N-nitrosourea alkylating drug carmustine/BCNU

Anticancer active *N*-nitrosoureas were developed in the course of a screening program initiated by the National Cancer Institute. Starting from the hit compound 1-methyl-3-nitro-1-nitrosoguanidine further analogs were prepared leading to 1-methyl-1-nitrosourea which was active against intracerebrally implanted murine leukemia^[105]^. Further fine-tuning of this compound finally led to carmustine/BCNU (bis-chloroethylnitrosourea, Figure 3) which entered clinical trials in 1964 and was approved by the FDA in 1977 for the treatment of brain tumors (BCNU passes the blood-brain-barrier because of its lipophilicity), lymphomas and myeloma^[106]^. A newer study recommends the application of BCNU for the treatment of recurrent GBM^[107]^. BCNU is a prodrug, which decomposes to afford alkylating chloroethyl moieties that can form DNA interstrand crosslinks^[108]^. Carbamoylation of nucleoprotein lysine residues via isocyanate intermediates can also play a role for the anticancer mode of action of BCNU^[109]^.

Expression analysis of BCNU-treated glioma cells led to dysregulation of let-7b (tumor suppressor), miR-125b-2 (oncomir), miR-133a-1 (tumor suppressor/oncomir), and miR-183 (oncomir)^[110]^. It was also shown that miR-21 expression induced BCNU-resistance in glioma cells via downregulation of Spry2 (sprout homolog 2)^[111]^. Although miR-181d was identified as a tumor suppressor and temozolomide-sensitizing factor (see above), GBM patients with implanted BCNU wafers displayed prolonged overall and progression-free survival in case of suppressed miR-181d expression^[112]^. In addition, high expression of the oncomir miR-221 suppressed PTEN and led to PI3K/Akt activation and resistance to BCNU^[113]^. A list of miRNAs involved in BCNU anticancer activity is given in Table 11.

**Table 11:** MiRNAs with effects on the anticancer activity of BCNU

MiRNA	Target	Function	Expression in cancers/tissues
let-7b	-	Tumor suppressor	Dysregulated in glioma cells upon BCNU treatment
miR-21	Spry2	Oncomir	Expression in glioma cells induced resistance
miR-125b-2	-	Oncomir	Dysregulated in glioma cells upon BCNU treatment
miR-133-1	-	Oncomir/tumor suppressor	Dysregulated in glioma cells upon BCNU treatment
miR-181d	-	Oncomir	Suppression led to prolonged survival of GBM patients
miR-183	-	Oncomir	Dysregulated in glioma cells upon BCNU treatment
miR-221	PTEN	Oncomir	High expression in GBM induced resistance

PTEN: phosphatase and TENsin homolog; Spry2: sprouty homolog 2

## Natural alkylating agents and their interactions with miRNAs

**Figure 4:**
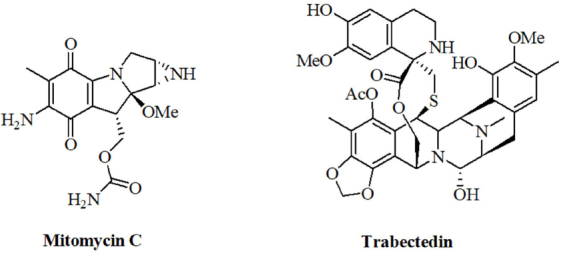
Structures of the natural alkylating agents mitomycin C and trabectedin

Natural alkylating agents were investigated for anticancer activity since the 1950’s^[114,115]^. Meanwhile, natural alkylating agents are widely applied for the therapy of various cancer diseases. The alkaloids mitomycin C and trabectedin were approved for the therapy of cancer [Figure 4]. The influence of miRNAs on the activity of these natural alkylating drugs is discussed below.

## Mitomycins, miRNAs and cancer

Mitomycins are bacterial indole alkaloids. The first mitomycins A and B were isolated in 1956 before mitomycin C was obtained as blue-violet crystals from *Streptomyces caespitosus* by Japanese groups in 1958^[116,117]^. Mitomycin C turned out to be the most anticancer active derivative of this group of mitomycins and entered clinical trials in Japan shortly after its discovery^[118]^. Currently, it is applied for the treatment of localized bladder cancer, anal cancer, head-and-neck cancer, and breast cancer (palliative 2nd or 3rd line treatment)^[119]^. Mitomycin C is a prodrug and activated mitomycin C forms DNA crosslinks that are highly lethal to cancer cells^[120]^. Activation of mitomycin C occurs via reduction of the benzoquinone scaffold to a hydroquinone (a reaction catalyzed by enzymes such as DT-diaphorase). Subsequent elimination of methanol forms a reactive aziridine system, which reacts with DNA via aziridine ring opening. Elimination of carbamate enables a second alkylating step leading to DNA crosslinking^[120]^.

Transfection of HT-29 colon cancer cells with let-7-1 suppressed COP9 signalosome expression and increased mitomycin C activity^[121]^. Expression of the tumor suppressor miR-31 enhanced the tumor growth inhibition of urothelial bladder cancer by mitomycin C both *in vitro* and *in vivo*^[122]^. MiR-31 inhibited integrin α5 directly and suppressed Akt and ERK signaling^[122]^. In addition, miR-34a expression sensitized medulloblastoma to mitomycin C treatment by downregulation of melanoma antigen A and upregulation of p53^[123]^. In contrast to that, inhibition of miR-191-5p expression led to higher mitomycin C activity against breast cancer cells via induction of apoptosis, SRY-box 4, and p53^[124]^. A slight increase in mitomycin C resistance was observed for Snail-expressing mesenchymal MCF-7 breast cancer cells, which displayed suppressed miR-200 family expression^[125]^. Mitomycin C was shown to induce senescence in human mesenchymal stem cells via upregulation of the tumor suppressor aminoacyl-tRNA synthetase-interacting multifunctional protein 3/p18, and suppression of AIMP3/p18 was observed for miR-543 and miR-590-3p^[126]^. This mechanism may also play a role for the anticancer activity of mitomycin C. Oxaliplatin-resistant HCT-116/l-OHP colon cancer cells transfected with miR-1915 mimics revealed enhanced mitomycin C sensitivity by suppression of Bcl-2^[127]^. A list of miRNAs involved in mitomycin C anticancer activity is given in Table 12.

**Table 12:** MiRNAs with effects on the anticancer activity of mitomycin C

MiRNA	Target	Function	Expression in cancers/tissues
let-7-1	CSN	Tumor suppressor	Transfection sensitized colon cancer cells
miR-31	ITGA5	Tumor suppressor	Expression sensitized urothelial bladder cancer
miR-34a	MAGE-A, p53	Tumor suppressor	Expression sensitized medulloblastoma
miR-191-5p	SOX4	Oncomir	Suppression sensitized breast cancer
miR-200	Zeb1, Zeb2, Slug	Tumor suppressor	Suppression correlated with resistance of breast cancer cells
miR-543	AIMP3/p18	Oncomir	Expression in mesenchymal stem cells blocked senescence
miR-590-3p	AIMP3/p18	Oncomir	Expression in mesenchymal stem cells blocked senescence
miR-1915	Bcl-2	Tumor suppressor	Transfection sensitized colon cancer cells

AIMP3: aminoacyl-tRNA synthetase-interacting multifunctional protein 3; Bcl-2: B-cell lymphoma 2; CSN: COP9 signalosome; ITGA5: integrin α5; MAGE-A: melanoma antigen A; Slug: Snail homolog; Zeb1/2: zinc finger E-box homeobox 1/2; SOX4: SRY-box 4

Since mitomycin C is a DNA-damaging drug, its long-term genotoxic effects and the inheritable aberration of miRNA expression induced by mitomycin C were investigated. Indeed, the treatment of HeLa cells with mitomycin C exhibited upregulated inherited expression of oncomirs such as miR-19b-3p, miR-21-3p, miR-30a-3p, miR-30e-3p, and miR-182-5p as well as suppressed inherited expression of the tumor suppressors miR-23b-3p, miR-29b-3p, miR-99a-5p, miR-99b-5p, miR-100-5p, miR-148a-3p, miR-193a-3p,,iR-340-5p, and miR-365-3p^[128]^. It is possible that new tumors can form basing on the long-term effects of mitomycin C and the observed aberrant miRNA expression.

## Trabectedin, miRNAs and cancer

Trabectedin (ecteinascidin 743, Yondelis^®^) is a rather new natural alkylating agent that belongs to the class of tetrahydroisoquinoline alkaloids. It was isolated from the Caribbean tunicate *Ecteinascidia turbinata*, which is only the host of the trabectedin-producing symbiont *Endoecteinascidia frumentensis*^[129]^. The high anticancer activity of trabectedin led to its clinical approval for the treatment of soft tissue sarcoma^[129]^. The unique DNA-damaging mechanism of trabectedin includes binding to nitrogen-N2 of guanine DNA bases in the minor groove. This DNA-trabectedin adduct interacts with DNA repair proteins of the transcription-coupled nucleotide excision repair DNA-repair system which causes cell death via the formation of double-strand breaks mainly in homologous recombination-deficient cells^[129]^. This mechanism is almost unique among known alkylating agents and only illudins have revealed a similar mode of action^[130]^. Trabectedin also blocked the transcriptional activity of fused in sarcoma-C/EBP-homologous protein (FUS-CHOP)^[131]^. The difference in miRNA expression between trabectedin-sensitive and trabectedin-resistant myxoid liposarcoma cells (402-91 sensitive and 402-91/ET trabectedin-resistant cells) was investigated and the resistant cells showed two-fold higher miR-21 expression (target: PDCD4/programmed cell death 4) as well as three-fold lower let-7e expression (targets: CCND1, E2F5, SEMA4C)^[132]^. The oncomir miR-7 was also upregulated while the tumor suppressors miR-98, miR-130a and miR-192 were suppressed in the resistant 402-91/ET cells^[132]^. The miRNAs miR-7, miR-21 and miR-130a probably act via FUS-CHOP since these miRNAs have CHOP-binding motifs^[132]^. In cholangiocarcinoma, trabectedin treatment led to upregulation of the oncomir miR-494-3p^[133]^. In addition, the tumor suppressors let-7c and miR-214-3p were suppressed^[133]^. Interestingly, trabectedin downregulated the oncomirs miR-21-3p, miR-21-5p, and miR-331-3p (oncomir in HCC), and upregulated the tumor suppressors miR-375 (tumor suppressor in colon and pancreatic cancer) and miR-4284 (tumor suppressor in glioblastoma), which may be a reason for the relatively high activity of trabectedin in this cancer model^[133]^. A list of miRNAs involved in trabectedin anticancer activity is given in Table 13.

**Table 13:** MiRNAs with effects on the anticancer activity of trabectedin

MiRNA	Target	Function	Expression in cancers/tissues
let-7c	-	Tumor suppressor	Suppression in cholangiocarcinoma upon trabectedin treatment
let-7e	CCND1, E2F5, SEMA4C	Tumor suppressor	Suppression in 402-91/ET cells led to resistance
miR-7	FUS-CHOP	Oncomir	Upregulation in 402-91/ET cells led to resistance
miR-21	PDCD4	Oncomir	Upregulation in 402-91/ET cells led to resistance, suppression in cholangiocarcinoma upon trabectedin treatment
miR-98	-	Tumor suppressor	Suppression in 402-91/ET cells led to resistance
miR-130a	FUS-CHOP	Tumor suppressor	Suppression in 402-91/ET cells led to resistance
miR-192	-	Tumor suppressor	Suppression in 402-91/ET cells led to resistance
miR-214-3p	TWIST	Tumor suppressor	Suppression in cholangiocarcinoma upon trabectedin treatment
miR-331-3p	EMT	Oncomir	Suppression in cholangiocarcinoma upon trabectedin treatment
miR-375	PI3K/Akt	Tumor suppressor	Upregulation in cholangiocarcinoma upon trabectedin treatment
miR-494-3p	-	Oncomir	Upregulation in cholangiocarcinoma upon trabectedin treatment
miR-4284	-	Tumor suppressor	Upregulation in cholangiocarcinoma upon trabectedin treatment

CCND1: cyclin D1; E2F5: E2F transcription factor 5; EMT: epithelial-to-mesenchymal transition; FUS-CHOP: fused in sarcoma-C/EBP-homologous protein; PDCD4: programmed cell death 4; PI3K/Akt: phosphatidylinositol-4,5-bisphosphate 3-kinase/ak thymoma; SEMA4C: semaphoring-4C; TWIST: twist transcription factor

## Conclusion

Alkylating drugs still play a crucial role for the therapy of various cancer diseases. While some examples are only applied for the treatment of special tumors (e.g., estramustine for the treatment of prostate cancer), other drugs (e.g., cyclophosphamide) are widely applied. The anticancer activity of these alkylating agents is regulated by various cellular factors. Aside proteins, small RNA molecules called miRNAs revealed a crucial role for the outcome of therapies based on alkylating drugs. Vice versa, alkylating drugs can also regulate miRNA expression leading to enhanced sensitivity of the affected cancer. Thus, a detailed understanding of the interplay between alkylating drugs and miRNAs is crucial for the development of new and improved cancer therapies. In particular, combination therapies with alkylating agents should be carefully checked in view of the corresponding miRNAs involved in alkylating drug response and resistance, and the co-drugs for combination therapies with these alkylating agents should be selected accordingly. Prolonged survival and improved quality of life would be possible and conceivable prospects for many cancer patients.

## Declarations

### Copyright

© The Author(s) 2019.
